# Regadenoson Cardiac Stress Test-Induced Takotsubo Cardiomyopathy: A Case Report

**DOI:** 10.7759/cureus.8004

**Published:** 2020-05-07

**Authors:** Saira Farid, Muhammad Ahsan, Hector M Garcia-Garcia

**Affiliations:** 1 Internal Medicine, MedStar Washington Hospital Center - Georgetown University Hospital, Washington, D.C., USA; 2 Cardiology, Mercy Health - St. Vincent Medical Center, Toledo, USA; 3 Cardiology, MedStar Washington Hospital Center, Washington, D.C., USA

**Keywords:** regadenoson, cardiac stress test, takotsubo cardiomyopathy

## Abstract

A 79-year-old female presented with acute left-sided chest pain with shortness of breath; she was afebrile and vitally stable. She had a mildly elevated troponin (0.11 ng/mL). Her N terminal pro B-type natriuretic peptide (NT-proBNP) was 7053 pg/mL and electrocardiography (ECG) showed nonspecific ST, T wave changes. Transthoracic echocardiogram (TTE) revealed an ejection fraction (EF) of 65-70%. She was diagnosed with a non-ST elevation myocardial infarction (NSTEMI) and underwent a nuclear stress test, which was negative for ischemia with no left ventricular motion abnormality and an EF of 73%. The patient developed acute respiratory failure following the Lexiscan (Astellas Pharma US, Northbrook, IL) and had to be intubated. A chest X-ray showed pulmonary edema, and transesophageal echocardiography (TEE) revealed a severely reduced EF of 25% with a new anterior wall motion abnormality. Left heart catheterization showed no significant coronary artery disease. Ventriculogram revealed a significantly reduced EF of 30% with apical akinesia. These findings were compatible with myocardial infarction with non-obstructive coronary arteries (MINOCA), likely secondary to regadenoson, which presented like takotsubo cardiomyopathy (TCM). Her condition gradually improved and the follow-up echo revealed baseline EF without symptoms of heart failure. In conclusion, takotsubo cardiomyopathy can be a potential complication from Lexiscan and can present as new-onset heart failure after the stress test.

## Introduction

Takotsubo cardiomyopathy (TCM) is a cardiac syndrome that involves a transitory decrease in the ejection fraction (EF) in the absence of significant coronary artery disease and reversible left ventricular or mid ventricular apical akinesis [1]. The term takotsubo was coined in Japan, based on the resemblance of the heart to an octopus trap used in Japan, called ‘takotsubo’ in Japanese [2]. TCM is known to be commonly triggered by strong physical or emotional stress; however, it sometimes occurs without any identifiable stressors and mimics acute coronary syndrome (ACS) [3]. Patients often present with chest pain, have ST-segment elevation on electrocardiography (ECG), and show modestly elevated cardiac enzyme biomarkers consistent with myocardial infarction (MI). The exact pathophysiology is unknown; however, it is commonly proposed to be due to catecholamine surge causing temporary coronary vasospasm. In this report, we present a case of regadenoson cardiac stress test-induced TCM in a 79-year-old female patient.

## Case presentation

A 79-year-old female presented with acute, spontaneous, left-sided chest pain associated with shortness of breath and lightheadedness for the past four hours. The pain was continuous, non-radiating, and 4/10 in intensity, and it had started when she had been getting ready for bed. She was nauseated but denied emesis, diaphoresis, or palpitations. She denied any history of angina or having similar pain in the past. In the emergency department, she was afebrile and vitally stable but had elevated blood pressures of 163/75 mm Hg. Her medical history was significant for end-stage renal disease requiring hemodialysis, atrial fibrillation, hypertension, and sarcoidosis. Additionally, initial laboratory investigations revealed a mildly elevated troponin (0.11 ng/mL), N terminal pro B-type natriuretic peptide (NT-proBNP) at 7053 pg/mL, and nonspecific ST, T wave changes on ECG. Transthoracic echocardiogram (TTE) revealed an EF of 65-70%, severe dilatation of left atrium, with mitral annular calcification. These findings were consistent with her last echo 10 months back. She was diagnosed with a non-ST elevation myocardial infarction (NSTEMI) and scheduled for a nuclear stress test. She was given 0.4 mg regadenoson (Lexiscan, Astellas Pharma US, Northbrook, IL) tetrofosmin intravenously, and the test was negative for ischemia with no left ventricular motion abnormality and an EF of 73%. The patient experienced severe shortness of breath after the regadenoson injection and was given intravenous theophylline, which led to a transient improvement of her symptoms. However, her shortness of breath eventually worsened and she became tachycardic (141/min) and hypotensive (86/59 mm Hg); her oxygen saturation was not detectable on the pulse oximetry, and she was placed on Bilevel Positive Airway Pressure (BiPAP). She did not show any improvement and was subsequently intubated.

**Figure 1:**
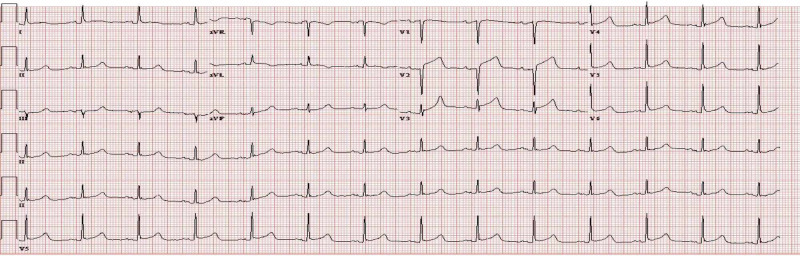
EKG at the time of the event EKG: electrocardiogram

**Figure 2:**
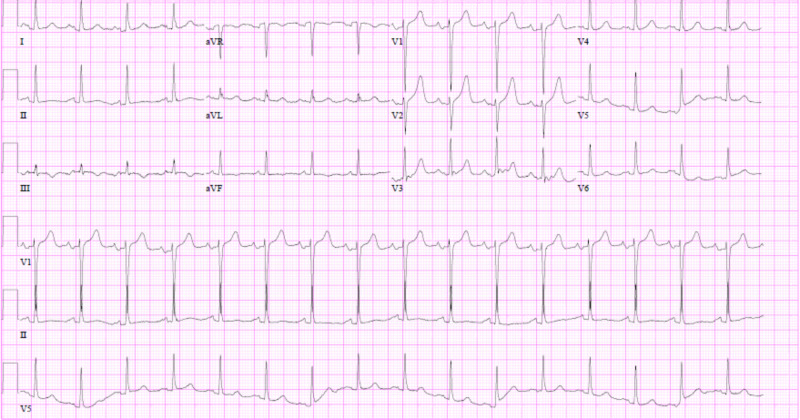
Baseline EKG EKG: electrocardiogram

**Figure 3:**
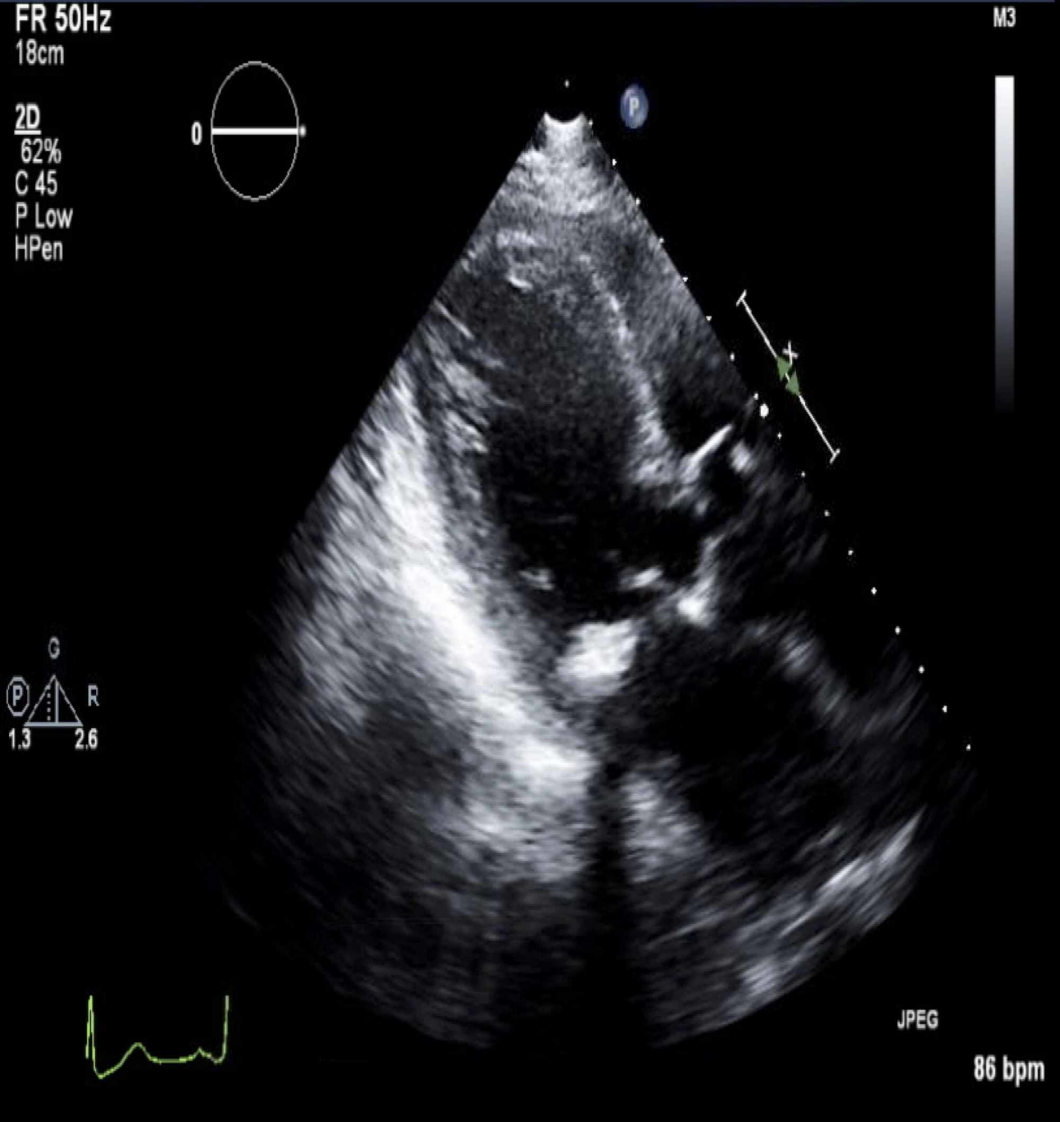
TEE showing left ventricle during diastole TEE: transesophageal echocardiography

**Figure 4:**
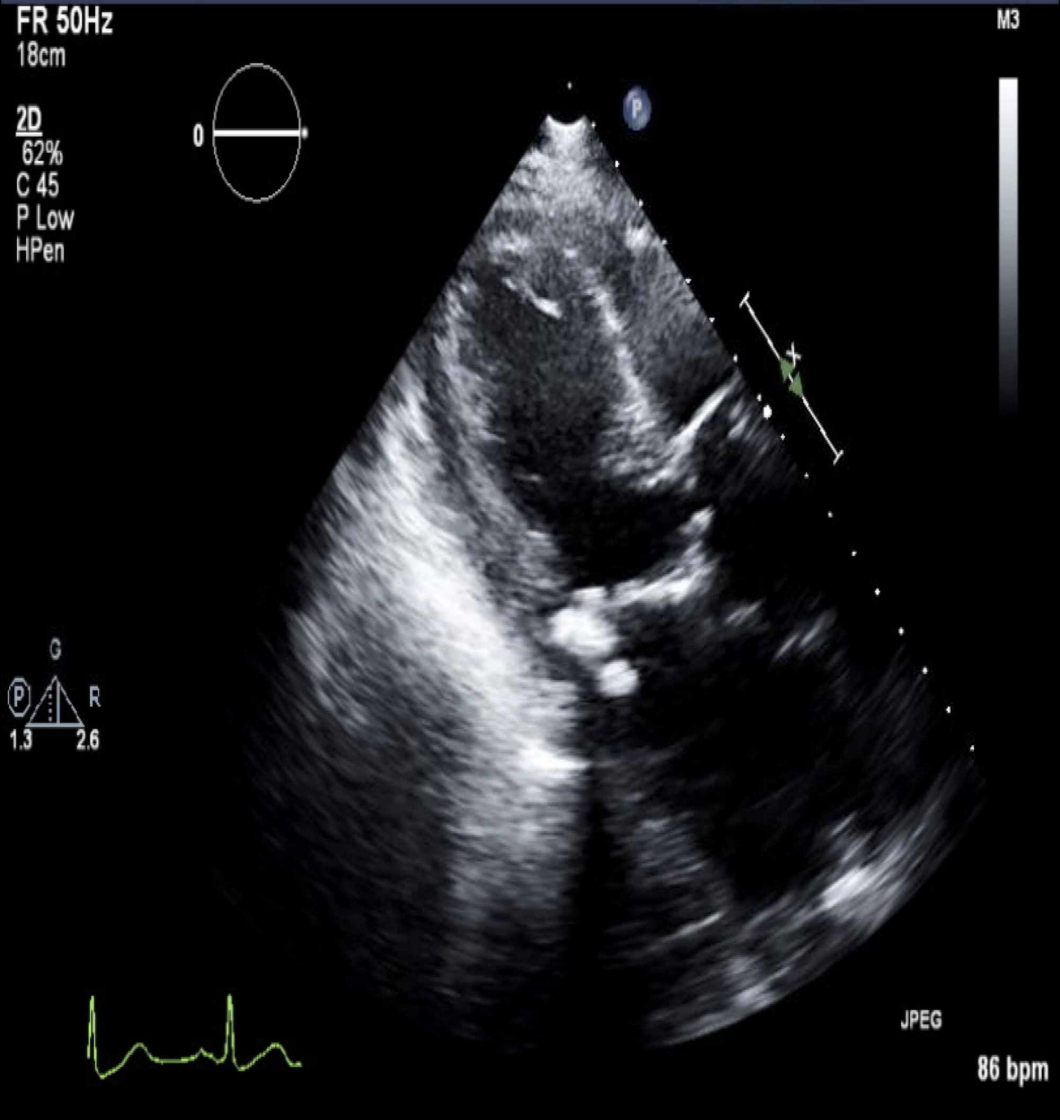
TEE with EF of 25% and a new anterior wall motion abnormality TEE: transesophageal echocardiography; EF: ejection fraction

On exam, while she was intubated, she was awake but lethargic with a Glasgow Coma Scale (GCS) of 5T. She had bilateral diffuse coarse crackles, and a cardiovascular exam revealed S1, S2, S4, and 1+ pitting pedal edema. Chest X-ray showed pulmonary edema secondary to acute systolic heart failure; she had a troponin level of 2.260 ng/mL and NT-proBNP up to 41,182. Her EKG revealed prolonged PR interval, early repolarization changes in V3-V6, and ST elevations in leads V2, V3, and V4, consistent with acute anterior MI (Figure 1), compared to her baseline EKG (Figure 2). Transesophageal echocardiography (TEE) revealed a severely reduced EF of 25% with a new wall motion abnormality, which was consistent with anterior wall MI (Figures 3, 4).

Subsequently, left heart catheterization was done, and it showed no significant coronary artery disease. Ventriculogram revealed a significantly reduced EF of 30% with apical akinesia. These findings were compatible with myocardial infarction with non-obstructive coronary arteries (MINOCA) thought to be secondary to takotsubo cardiomyopathy. She was continued on aspirin, statin, beta-blocker, and started on angiotensin-converting enzyme (ACE) inhibitor subsequently for her MI/heart failure (HF). Her condition gradually improved. She was extubated, her troponin trended down, and her EKG changes resolved. At the eight-week follow-up, she was asymptomatic, and a repeat echo revealed recovered EF of 60-65%.

## Discussion

TCM, also known as stress cardiomyopathy, is a rare but well-known entity and is estimated to account for up to 0.02% of all in-patient admissions [4]. It has been estimated that 89% of cases occur in women, typically post-menopause. Common co-morbid conditions include cerebrovascular disease, psychiatric disorders, chronic liver disease, sepsis, and malignancy [5,6]. The typical presentation of TCM is usually chest pain and dyspnea that mimics ACS. Patients also have associated transient EKG changes, elevated cardiac biomarkers, and wall motion abnormalities on echocardiogram [1].

The etiology of the condition is not completely understood but the most commonly accepted hypothesis is the endogenous catecholamine-induced myocardial stunning in the background of severe emotional or physiological stress [7]. It has been proposed that TCM may actually be a myocardial protective response elicited via a change in G-protein coupling (Gs to Gi subunit) by the beta2 adrenergic receptor, known as ‘signal trafficking’. Additionally, adrenergic receptor polymorphisms may predispose certain patients to TCM. TCM may be accompanied by coronary vasospasm, plaque rupture, and thrombus formation with spontaneous resolution. A large retrospective study done to analyze the safety of regadenoson’s use in stress testing did not reveal TCM [8,9]. However, other medications that have been described to cause TCM include epinephrine, atropine, dobutamine, and ephedrine, possibly due to the effect of these drugs on the sympathetic nervous system and could be attributed to the theory of catecholamine-induced cardiomyopathy [10,11].

Regadenoson is known to induce myocardial ischemia; however, TCM’s association with regadenoson is exceedingly rare. There is only one previously reported case of such association with a similar presentation of new-onset heart failure; this was reported in a middle-aged female following Lexiscan, with a clean left heart catheterization and spontaneous resolution of the heart failure [12]. Regadenoson is a selective adenosine A2A receptor agonist developed to reduce the adverse effects experienced with adenosine [8]. It has been broadly incorporated into clinical practice for cardiac perfusion imaging. FDA Adverse Event Reporting System (FAERS) has reported 26 cases of MI and 29 deaths following Lexiscan, occurring mostly within six hours following the test [13]. The mechanism by which regadenoson causes coronary vasospasm and infarction is unknown. The prognosis in TCM is typically excellent, and most patients recover completely within four to eight weeks.

## Conclusions

In conclusion, takotsubo cardiomyopathy can be a potential complication of the Lexiscan stress test. It is important to consider this and recognize the association between new-onset heart failure and recent Lexiscan stress test. Patients can present with delayed symptoms of new-onset heart failure after the stress test. Hence, even an uncomplicated Lexiscan still carries a risk.
